# Cell-free DNA 5-hydroxymethylcytosine is highly sensitive for MRD assessment in acute myeloid leukemia

**DOI:** 10.1186/s13148-023-01547-0

**Published:** 2023-08-24

**Authors:** Jianming Shao, Shilpan Shah, Siddhartha Ganguly, Youli Zu, Chuan He, Zejuan Li

**Affiliations:** 1 Department of Pathology and Genomic Medicine, Houston Methodist Hospital, Houston, TX 77030 USA; 2 Neal Cancer Center, Houston Methodist Hospital, Houston, TX 77030 USA; 3 Weill Cornell Medical College, New York, NY 10065 USA; 4 Houston Methodist Research Institute, Houston, TX 77030 USA; 5 Department of Chemistry, Department of Biochemistry and Molecular Biology, Institute for Biophysical Dynamics, The University of Chicago, Chicago, IL 60637 USA; 6 Howard Hughes Medical Institute, The University of Chicago, Chicago, IL 60637 USA

**Keywords:** Cell-free DNA, 5-hydroxymethylcytosine, Acute myeloid leukemia, Measurable residual disease

## Abstract

Measurable residual disease (MRD) is an important biomarker in acute myeloid leukemia (AML). However, MRD cannot be detected in many patients using current methods. We developed a highly sensitive 5-hydroxymethylcytosine (5hmC) signature in cell-free DNA by analyzing 115 AML patients and 86 controls. The 5hmC method detected MRD in 20 of 29 patients with negative MRD by multiparameter flow cytometry and 11 of 14 patients with negative MRD by molecular methods. MRD detection by the 5hmC method was significantly associated with relapse-free survival. This novel method can be used in most AML patients and may significantly impact AML patient management.

**Supplementary Information:**

The online version contains supplementary material available at 10.1186/s13148-023-01547-0.

## Introduction

Acute myeloid leukemia (AML) is characterized by epigenetic aberrations that arise early in cancer development [1]. Though most AML patients achieve remission after initial induction therapy [2], leukemia relapse occurs in over 50% of patients within two to three years of initial treatment [2]. Early detection of measurable residual disease (MRD) significantly impacts patient management and therapeutic decisions [2]. MRD refers to the low levels of residual leukemia that cannot be detected by morphologic assessment alone [3]. Detection of MRD is strongly associated with adverse outcomes in AML patients and is an important prognostic and predictive marker to refine risk assessment and inform treatment decision-making [3].

Currently, assessment of MRD is based on leukemia-associated immunophenotypes by multiparameter flow cytometry (MFC) and genotypic aberrations by molecular methods, such as reverse transcriptase-quantitative PCR (RT-qPCR) and next-generation sequencing (NGS) [2, 3]. Though MFC is applicable in > 90% of AML patients, it has a lower sensitivity than RT-PCR and requires a high level of user expertise [2, 3]. Despite the high sensitivity of molecular MRD methods, only a small portion of patients have suitable genetic alterations for monitoring [2, 3]. With current MRD assessments, the five-year overall survival (OS) is 68% for AML patients with a negative MRD and 34% for patients with a positive MRD [4], indicating that many patients with negative MRD may benefit from a more sensitive MRD marker.

5-hydroxymethylcytosine (5hmC) is an emerging DNA marker in cancer and the first intermediate product in the oxidation of 5-methylcytosine (5mC) by ten-eleven-translocation proteins [5, 6]. It is significantly correlated with gene expression and 5hmC levels change early and dynamically in cancer [5, 6]. Specifically, a global decrease in 5hmC occurs in AML and many other malignancies and correlates with somatic mutations or abnormal expression in DNA-methylation-related genes in AML patients [5–7]. A high level of 5hmC is associated with adverse OS in AML [7]. Using a highly sensitive nano-hmC-Seal method, we and others have demonstrated that plasma cell-free DNA (cfDNA) 5hmC is highly sensitive for the detection and prognosis of AML and other malignancies [5, 6, 8, 9]. Thus, we hypothesized that cfDNA 5hmC could act as a highly sensitive marker of MRD in AML.

Here we demonstrate the utility of cfDNA 5hmC for MRD assessment using a highly specific nano-5hmC-Seal method in combination with NGS (nano-hmC-Seal-Seq) [10]. We profiled genome-wide 5hmC distribution in plasma cfDNA samples from 115 AML patients (Table S1) and 86 non-cancer individuals. Using improved analysis methods from our previous study [8], we developed a cfDNA 5hmC signature for MRD detection in AML in 86 patients. We then evaluated the 5hmC signature in 29 samples that lacked MRD by MFC. The 5hmC signature detected MRD in patients who were assessed as MRD negative by MFC and molecular methods. We observed that positive MRD detection using the 5hmC marker was significantly associated with shorter relapse-free survival (RFS), indicating that cfDNA 5hmC is a sensitive marker for MRD assessment in AML.

## Methods

Blood samples from 115 patients diagnosed with AML (Table S1) and 86 age and sex-matched non-cancer individuals were collected between 2005 and 2021 at Houston Methodist Hospital. One hundred and nine patients received chemotherapy and 57 received hematopoietic stem cell transplantation. Four patients did not receive any treatment and two did not have treatment information on record. For MRD detection, bone marrow samples were analyzed by MFC (71 samples) and molecular methods (56 samples), including NGS panels, *NPM1* RT-PCR, and *RUNX1-RUNX1T1* RT-PCR (Additional file 1: Table S1). All MFC and molecular methods for MRD detection were performed through standard clinical care. RFS was defined as the time of registration to failure to achieve complete remission (CR), relapse, loss of follow-up, or death as a result of any cause.

Plasma cfDNA extraction, library preparation, and NGS were performed as previously described [8, 9]. High-quality reads were counted into gene bodies (RefSeq) using featureCounts. We normalized raw gene read counts using counts per million (CPM) and 21,528 genes remained in reads with CPM ≥ 5 in more than half of the samples.

The plasma cfDNA 5hmC signature to detect AML was developed as previously described [8]. Briefly, we randomly split AML samples (excluding samples in CR) and control samples into a training set (40 AML and 40 control samples), a validation set (25 AML and 25 control samples), and a test set (21 AML and 21 control samples). We performed univariate logistic regression analysis adjusted for age and sex in 21,528 genes and obtained 8066 informative genes with a cutoff *P* < 0.05 in the training set. To select the high confidence markers, the elastic net model was cross-validated for a grid of parameter values of α (α range: 0.55–0.95 with 0.1 increments) using glmnet. This selection process was repeated 100 times and a list of 13 genes cross-validated in over 95% of sampling times was selected for the final weighted model. We then applied a multivariate logistic regression model to calculate the regression coefficient for each of the 13 genes. We then calculated a weighted-detection score (wd-score) for each sample in the training, validation, and test sets and in patients who had negative MRD by MFC analysis. Wd-score $={\sum }_{\mathrm{k}=1}^{\mathrm{n}}\left({\upbeta }_{\mathrm{k}}\times {\mathrm{G}}_{\mathrm{k}}\right)$. β_k_ is the coefficient from the multivariate logistic regression analysis for gene k, and G_k_ is the normalized 5hmC read counts of the k^th^ marker gene. The area under the curve and 95% confidence interval were calculated to evaluate model performance using pROC. A cutoff score simultaneously maximized with sensitivity and specificity was determined using optimal.cutpoints in the training set. Sensitivity and specificity were calculated based on the cutoff wd-score in the validation and test sets.

We performed plotting and statistical tests using R language version 4.1.1. Kaplan–Meier curves were used to display the RFS of patient groups categorized based on the wd-scores. The log-rank test was used to evaluate the statistical significance of RFS between groups. Comparisons of wd-scores between groups were analyzed using the Wilcoxon rank-sum test. A *P* value < 0.05 was considered significant.

## Results

**Fig. 1:**
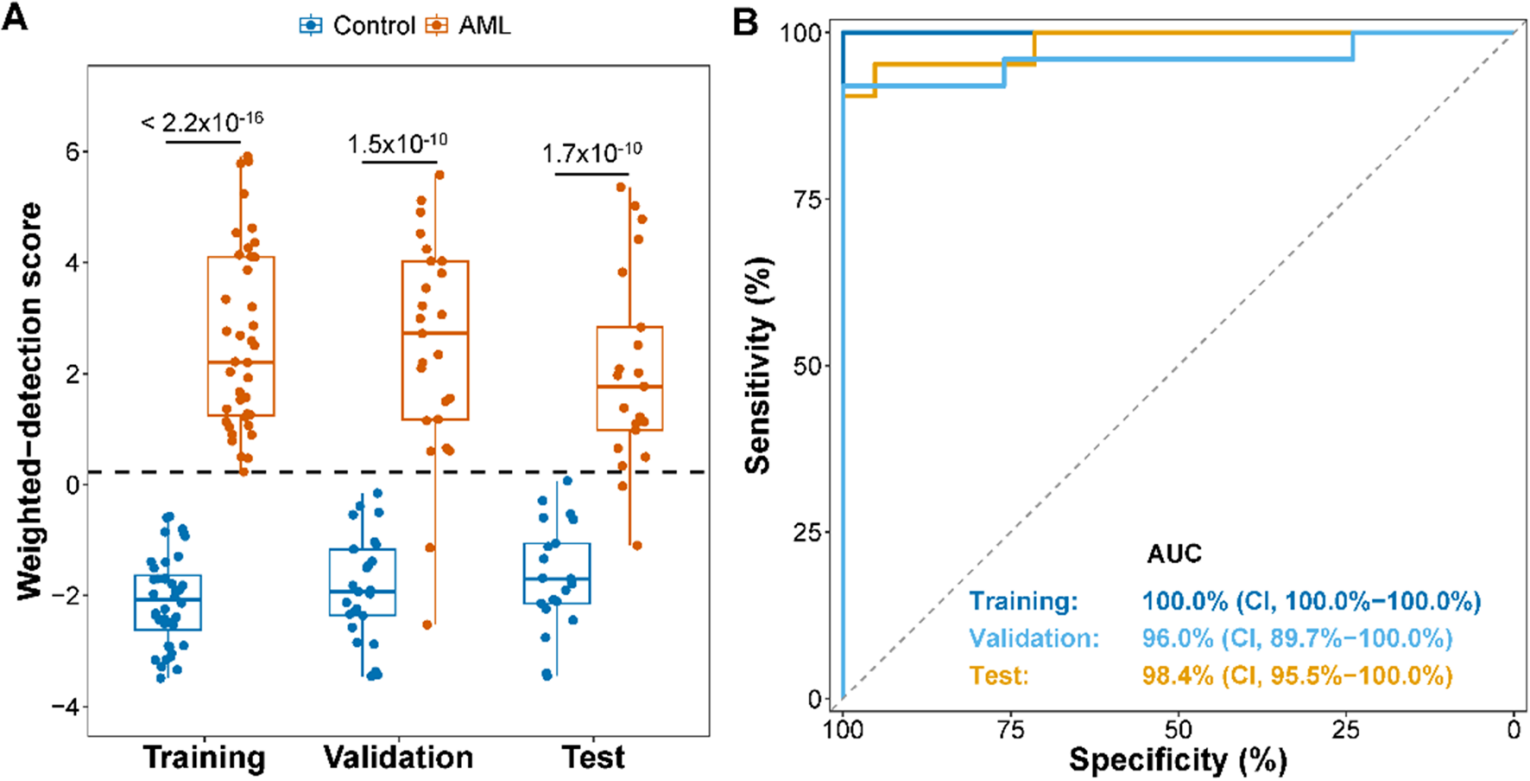
A cfDNA 5hmC signature differentiates AML patients from controls. **A** Boxplot of weighted-detection scores (wd-scores) in controls and AML samples in training, validation, and test sets. Black dashed line represents a cutoff score of 0.229. Center line represents median, bounds of box represent 25th and 75th percentiles, and whiskers are Tukey whiskers. **B** Receiver Operating Characteristics analysis of wd-score calculated from the 5hmC signature in the training, validation, and test sets. AUC, area under the curve. CI, 95% confidence interval

To develop a 5hmC signature to detect AML, we compared the 5hmC distribution between AML patients and controls and performed feature selection on differentially hydroxymethylated genes in the training set. We identified a 5hmC signature of 13 genes that accurately differentiated AML samples from controls (Additional file 1: Table S2). We then developed a weighted model and calculated a wd-score for each sample. The wd-scores calculated based on the 5hmC signature were significantly higher in AML patients compared to controls in the training (*P* < 2.2 × 10^–16^), validation (*P* = 1.5 × 10^–10^), and test (*P* = 1.7 × 10^–10^) sets (Fig. 1A). With a specificity of 100.0%, the sensitivity of the signature was 100.0%, 92.0%, and 90.5%, respectively, in the three sets (Additional file 1: Table S3). The area under the curve (AUC) was 100.0% [95% confidence interval (CI), 100.0%–100.0%], 96.0% (95% CI, 89.7%–100.0%), and 98.4% (95% CI, 95.5%–100.0%), respectively (Fig. 1B). Multivariate Cox proportional hazards model analysis showed that the 5hmC signature was independent of age and sex (Additional file 1: Table S4).

**Figure. 2:**
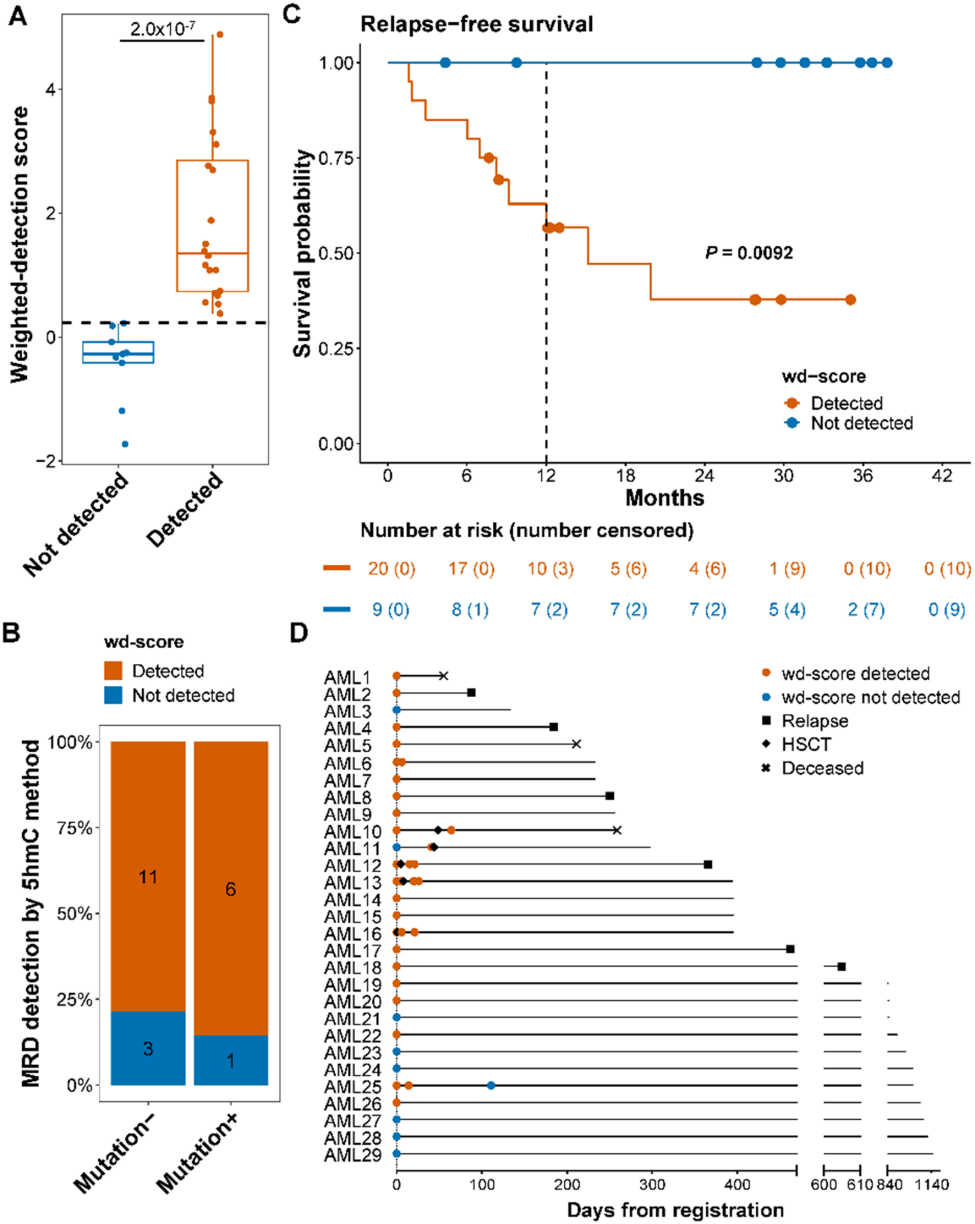
The cfDNA 5hmC signature is highly sensitive for measurable residual disease detection in AML. **A** Boxplot of weighted-detection scores (wd-scores) in AML samples with no measurable residual disease (MRD) assessed by multiparameter flow cytometry (MFC). Black dashed line represents a cutoff score of 0.229. Center line represents median, bounds of box represent 25th and 75th percentiles, and whiskers are Tukey whiskers. **B** Comparison of MRD detection by the cfDNA 5hmC method and molecular methods. Molecular-, no MRD detected by molecular methods. Molecular+, MRD detected by molecular methods. The number of patients is displayed on the columns. **C** Kaplan–Meier analysis of relapse-free survival (RFS) of AML patients with no MRD by MFC based on the cfDNA 5hmC method. Censored patients are indicated by dots. **D** Timing and MRD results were assessed using the cfDNA 5hmC method for each AML patient. AML patient samples with no MRD detected by MFC are displayed. Patients were ordered by total days of clinical follow-up from registration. HSCT, hematopoietic stem cell transplant

To evaluate the cfDNA 5hmC signature for MRD detection, we calculated wd-scores for 29 samples that lacked MRD by MFC. The cfDNA 5hmC method detected MRD in 20 of the 29 samples (Fig. 2A, Additional file 1: Table S5). The wd-scores were significantly higher in patients with MRD detection compared to the patients without MRD by the cfDNA 5hmC method (*P* = 2.0 × 10^–7^; Fig. 2A). Among the 29 samples, 21 were also evaluated by molecular methods, (Additional file 1: Table S6). The cfDNA 5hmC method showed a concordance of 85.7% (6 of 7) in molecular-positive MRD samples (Fig. 2B). The cfDNA 5hmC method also detected MRD in 11 (78.6%) of the 14 molecular-negative samples (Fig. 2B). Among the 11 cases that were MRD-negative by both MFC and molecular methods, but MRD-positive by the cfDNA 5hmC method, 8 were evaluated by NGS, 2 by *NPM1* analysis, and 1 by *RUNX1-RUNX1T1* analysis (Additional file 1: Table S5).

MRD assessment by the cfDNA 5hmC method was significantly associated with outcomes in AML patients. Relative to undetected MRD (low wd-scores), detected MRD (high wd-scores) was significantly associated with a shorter RFS (median 10.6 versus 31.6 months; *P* = 0.0092; hazard ratio 1.2 × 10^9^; 95% CI: 0 – infinite; Fig. 2C) in patients assessed by the cfDNA 5hmC method. The 12-month RFS rate was 100.0% in patients without MRD and 62.9% in patients with MRD detected (Fig. 2C). The association between MRD detection by the cfDNA 5hmC method and early disease relapse in individual patients is shown in Fig. 2D.

## Discussion

To improve MRD detection in AML, we developed a plasma cfDNA 5hmC signature that is highly sensitive for MRD assessment in AML patients. We based our approach on the cost-effective nano-hmC-Seal procedure, which captures whole gene expression changes from cfDNA [10]. The cfDNA 5hmC method was able to detect MRD in 20 of 29 patients who were MRD negative by MFC and molecular methods. MRD detection by the cfDNA 5hmC method was also significantly associated with clinical outcomes. As the sensitivity for molecular methods is as high as 0.001–0.0001% for RT-qPCR and approximately 0.1% for NGS [2], our cfDNA 5hmC signature may achieve a similar or higher sensitivity. Further investigation of the limit of detection is warranted.

Relative to current MRD detection methods, the cfDNA 5hmC signature has several advantages. First, assessment by cfDNA 5hmC reduces the need for painful bone marrow biopsy procedures and provides opportunities for more frequent and real-time monitoring of AML patients. In contrast, bone marrow is typically required for MFC and molecular assessment. Second, the cfDNA 5hmC method likely has a higher sensitivity than MFC and molecular methods. The increased sensitivity of 5hmC method is partly due to high abundance of 5hmC relative to immunophenotypic markers and gene mutations [3, 5]. Third, the cfDNA 5hmC signature is applicable to almost all patients, as epigenetic aberrations occur widely and commonly in cancer patients. Molecular assessment can only be applied to a select number of recurrent genetic changes in AML patients with a mutation [2, 3]. Fourth, the cfDNA 5hmC method does not require knowledge of prior epigenetic status. Conversely, immunophenotype or mutation information is usually required for MFC and molecular methods. Finally, the cfDNA 5hmC method is independent of germline mutations or clonal hematopoiesis, which may affect the accuracy of molecular methods. Therefore, the cfDNA 5hmC method may be a useful complement to molecular and MFC assessment for MRD.

Our study has some limitations. Notably, 24 of the 29 samples with no MRD by MFC were collected after hematopoietic stem cell transplant. Though we observed that the cfDNA 5hmC method was effective in these patients, additional evaluation of this method in pre-transplant patients is needed. Moreover, a larger-scale multicenter study would further demonstrate the utility of the cfDNA 5hmC method for MRD detection in AML. Compared to our previous study [8], we used a new normalization method that allows samples to be analyzed individually to reduce bias among batches. This method can be easily adapted to clinical scenarios where patient samples are analyzed independently.

In summary, we developed a highly sensitive blood marker for MRD assessment in AML. The cfDNA 5hmC method can detect MRD in patients who test MRD negative by MFC and molecular methods. Because 5hmC changes dynamically in almost all AML patients [7], the new method has the potential to reflect real-time disease status in most AML patients. Establishing new markers that accurately detect MRD will improve clinical management of AML patients and may dramatically improve clinical outcomes. The data described herein will provide a solid foundation for future clinical studies. The cfDNA 5hmC method provides a safe, easy, and minimally invasive approach for MRD detection and underscores the potential of investigatory epigenetics using cfDNA in leukemia and other hematological malignancies.

### Supplementary Information

**Additional file 1**. Tables S1–S6.

## Data Availability

The raw 5hmC sequencing data are available in the National Center for Biotechnology Information Gene Expression Omnibus database**.**
