# Supplementary material for: Cell-free DNA 5-hydroxymethylcytosine is highly sensitive for MRD assessment in acute myeloid leukemia
Source: Clin Epigenetics. 2023 Aug 24;15:134. doi: 10.1186/s13148-023-01547-0 (PMC10464230; doi:10.1186/s13148-023-01547-0)
Supplement: Supplementary file 1 — Additional file 1. Tables S1–S6. [file 13148_2023_1547_MOESM1_ESM.docx]

**Cell-free DNA 5-hydroxymethylcytosine is highly sensitive for MRD assessment in acute myeloid leukemia**

Jianming Shao, Shilpan Shah, Siddhartha Ganguly, Youli Zu, Chuan He, Zejuan Li

Table S1. Characteristics of the AML patients. 2

Table S2. List of the 5hmC signature genes. 3

Table S3. Sensitivity and Specificity of the 5hmC signature. 4

Table S4. Multivariate logistic regression analysis. 4

Table S5. Measurable residual disease status assessed by different methods. 5

Table S6. Results of molecular analyses. 6

# Table S1. Characteristics of the AML patients.

|  | **Training**  (n = 40) | **Validation**  (n = 25) | **Test**  (n = 21) | **In remission by MFC** (n = 29) | **All**  (N = 115) |
| --- | --- | --- | --- | --- | --- |
| **Sex (male/female)** | 25/15 | 13/12 | 11/10 | 15/14 | 64/51 |
| **Age, (median, range, y)** |  |  |  |  |  |
| ≥60 | 17 | 17 | 13 | 12 | 59 |
| <60 | 23 | 8 | 8 | 17 | 56 |
| **Treatment** |  |  |  |  |  |
| No treatment | 4 | 0 | 0 | 0 | 4 |
| Chemotherapy | 34 | 25 | 21 | 29 | 109 |
| HSCT | 15 | 4 | 11 | 27 | 57 |
| No treatment info | 2 | 0 | 0 | 0 | 2 |
| **MFC, blasts in BM** |  |  |  |  |  |
| Detected | 0 | 21 | 21 | 0 | 42 |
| Undetected | 0 | 0 | 0 | 29 | 29 |
| Not available | 40 | 4 | 0 | 0 | 44 |
| **Cytogenetics** |  |  |  |  |  |
| Normal | 13 | 11 | 8 | 19 | 51 |
| Abnormal | 15 | 13 | 11 | 10 | 49 |
| Unknown | 12 | 1 | 2 | 0 | 15 |
| **Gene mutations**  (positive/total No. of patients tested) | 1/2 | 18/18 | 14/15 | 7/21 | 40/56 |
| *NPM1* | 1/2 | 4/17 | 2/15 | 1/20 | 8/54 |
| *RUNX1-RUNX1T1* | 0/0 | 0/0 | 0/0 | 0/1 | 0/1 |
| NGS panel | 1/2 | 18/18 | 14/15 | 7/20 | 40/55 |

HSCT, hematopoietic stem cell transplant; y, year; MFC, multiparameter flow cytometry; BM, bone marrow; NGS, next-generation sequencing.

# Table S2. List of the 5hmC signature genes.

| **Gene name** | **Chr** | **Start** | **End** | **Strand** | **Coefficient** |
| --- | --- | --- | --- | --- | --- |
| ***SLC35E2A*** | chr1 | 1724838 | 1746070 | - | 0.439022 |
| ***LOC107985792*** | chr2 | 23018105 | 23198929 | - | -0.63156 |
| ***LINC01206*** | chr3 | 181952364 | 182010678 | + | -0.39865 |
| ***PLAAT1*** | chr3 | 193241128 | 193281639 | + | -0.47153 |
| ***LOC101927378*** | chr7 | 84532476 | 84584318 | + | 0.545482 |
| ***PRSS37*** | chr7 | 141836278 | 141849725 | - | -0.46995 |
| ***LOC105375856*** | chr8 | 57855478 | 57958514 | + | -0.58944 |
| ***CNTNAP3B*** | chr9 | 41890536 | 42129426 | - | 0.476893 |
| ***LOC107987088*** | chr9 | 85084727 | 85142635 | - | -0.58279 |
| ***LOC105376436*** | chr10 | 17437290 | 17446781 | + | 0.575624 |
| ***TRPC6*** | chr11 | 101451470 | 101584007 | - | -0.34683 |
| ***ATP10A-DT*** | chr15 | 25865284 | 25877190 | + | 0.509099 |
| ***LINC02597*** | chr20 | 45178478 | 45191638 | + | -0.61703 |

Chr, chromosome.

# Table S3. Sensitivity and Specificity of the 5hmC signature.

| **Training** | | | **Validation** | | **Test** | |
| --- | --- | --- | --- | --- | --- | --- |
|  | **AML** | **Control** | **AML** | **Control** | **AML** | **Control** |
| **wd-score high** | 40 | 40 | 23 | 25 | 19 | 21 |
| **wd-score low** | 0 | 0 | 2 | 0 | 2 | 0 |
| **Total** | 40 | 40 | 25 | 25 | 21 | 21 |
| **Sensitivity** | 100.0% | - | 92.0% | - | 90.5% | - |
| **Specificity** | - | 100.0% | - | 100.0% | - | 100.0% |

# Table S4. Multivariate logistic regression analysis.

| **Training (n = 80)** | | **Validation (n = 50)** | **Test (n = 42)** |
| --- | --- | --- | --- |
| **wd-score** | < 2.0×10^-16^ | 1.2×10^-13^ | 1.7×10^-10^ |
| **Age** | 0.38 | 0.15 | 0.16 |
| **Sex** | 0.016 | 0.98 | 0.22 |

Multivariate logistic regression analysis was performed in 86 AML patients and 86 controls, considering wd-score, age, and sex. Wd-score, weighted detection score. The number of AML and control samples were equal for all groups.

# Table S5. Measurable residual disease status assessed by different methods.

| **ID** | **5hmC** | **MFC** | **Molecular methods** |
| --- | --- | --- | --- |
| AML1 | + | - | NA |
| AML15 | + | - | NA |
| AML17 | + | - | NA |
| AML2 | + | - | NGS - |
| AML7 | + | - | NGS - |
| AML13 | + | - | NGS - |
| AML14 | + | - | NGS - |
| AML16 | + | - | NGS - |
| AML19 | + | - | NGS - |
| AML22 | + | - | NGS - |
| AML26 | + | - | NGS - |
| AML4 | + | - | NGS + |
| AML6 | + | - | NGS + |
| AML8 | + | - | NGS + |
| AML9 | + | - | NGS + |
| AML10 | + | - | NGS + |
| AML12 | + | - | NGS + |
| AML5 | + | - | NPM1 - |
| AML20 | + | - | NPM1 - |
| AML18 | + | - | RUNX1-RUNX1T1 - |
| AML21 | - | - | NA |
| AML24 | - | - | NA |
| AML27 | - | - | NA |
| AML28 | - | - | NA |
| AML29 | - | - | NA |
| AML11 | - | - | NGS - |
| AML23 | - | - | NGS - |
| AML25 | - | - | NGS - |
| AML3 | - | - | NGS & NPM1 + |

Twenty-nine samples that lacked measurable residual disease (MRD) by multiparameter flow cytometry were displayed. MFC, multiparameter flow cytometry. NGS, next-generation sequencing. +, MRD positive. -, MRD negative. NA, not available.

| **ID** | **RT-PCR** | | **NGS panel** | | | | | | | | | | | | | | | | | | | |
| --- | --- | --- | --- | --- | --- | --- | --- | --- | --- | --- | --- | --- | --- | --- | --- | --- | --- | --- | --- | --- | --- | --- |
|  | **NPM1 insertion** | **RUNX1-RUNX1T1** | **TET2** | **IDH1** | **IDH2** | **TP53** | **DNMT1** | **DNMT3A** | **ASXL1** | **CEBPA** | **RUNX1** | **SRSF2** | **SETBP1** | **STAG2** | **JAK2** | **KIT** | **SF3B1** | **PTPN11** | **SH2B3** | **CUX1** | **GNAS** | **BCOR** |
| AML2 | - | NA | - | - | - | - | - | - | - | - | - | - | - | - | - | - | - | - | - | - | - | - |
| AML3 | + | NA | - | - | - | - | - | c.845del,  p.Pro282fs | - | - | - | - | - | - | - | - | - | - | - | - | - | - |
| AML4 | - | NA | - | - | - | - | c.833C>T,  p.Pro278Leu | - | - | - | - | - | - | - | - | - | - | - | - | - | - | c.5234A>C,  p.His1745Pro |
| AML5 | - | NA | NA | NA | NA | NA | NA | NA | NA | NA | NA | NA | NA | NA | NA | NA | NA | NA | NA | NA | NA | NA |
| AML6 | - | NA | - | - | - | - | - | c.2645G>A,  p.Arg882His | - | - | - | - | - | - | - | - | - | - | - | - | c.602G>A,  p.Arg201His | - |
| AML7 | - | NA | - | - | - | - | - | - | - | - | - | - | - | - | - | - | - | - | - | - | - | - |
| AML8 | - | NA | - | - | - | - | - | c.2645G>A,  p.Arg882His | - | - | - | - | - | - | - | - | - | - | - | - | - | - |
| AML9 | - | NA | - | - | - | - | - | - | - | - | - | - | - | - | - | - | - | - | c.894G>A,  p.Trp298* | c.1515G>A,  p.Met505Ile | - | - |
| AML10 | - | NA | c.4133G>A, p.Cys1378Tyr;  c.3823G>A, p.Gly1275Arg | - | - | - | - | - | c.1934dup,  p.Gly646fs | - | c.735dup,  p.Thr246fs | c.284C>A,  p.Pro95His | c.2608G>A,  p.Gly870Ser | c.347_359del,  p.Asp116fs | c.1849G>T,  p.Val617Phe | - | - | - | - | - | - | - |
| AML11 | - | NA | - | - | - | - | - | - | - | - | - | - | - | - | - | - | - | - | - | - | - | - |
| AML12 | - | NA | - | - | - | - | - | c.915G>A,  p.Trp305* | - | - | - | - | - | - | - | - | - | - | - | - | - | - |
| AML13 | - | NA | - | - | - | - | - | - | - | - | - | - | - | - | - | - | - | - | - | - | - | - |
| AML14 | - | NA | - | - | - | - | - | - | - | - | - | - | - | - | - | - | - | - | - | - | - | - |
| AML16 | - | NA | - | - | - | - | - | - | - | - | - | - | - | - | - | - | - | - | - | - | - | - |
| AML18 | - | NA | NA | - | - | NA | NA | NA | NA | NA | NA | NA | NA | NA | NA | NA | NA | NA | NA | NA | NA | NA |
| AML19 | - | NA | - | - | - | - | - | - | - | - | - | - | - | - | - | - | - | - | - | - | - | - |
| AML20 | NA | - | NA | NA | NA | NA | NA | NA | NA | NA | NA | NA | NA | NA | NA | NA | NA | NA | NA | NA | NA | NA |
| AML22 | - | NA | - | - | - | - | - | - | - | - | - | - | - | - | - | - | - | - | - | - | - | - |
| AML23 | - | NA | - | - | - | - | - | - | - | - | - | - | - | - | - | - | - | - | - | - | - | - |
| AML25 | - | NA | - | - | - | - | - | - | - | - | - | - | - | - | - | - | - | - | - | - | - | - |
| AML26 | - | NA | - | - | - | - | - | - | - | - | - | - | - | - | - | - | - | - | - | - | - | - |

**Table S6. Results of molecular analyses.**
